# Trajectories of Nutritional Quality, Diet-Related Environmental Impact, and Diet Cost in China: How Much Does Ultra-Processed Food and Drink Consumption Matter?

**DOI:** 10.3390/nu17020334

**Published:** 2025-01-17

**Authors:** Zhiyao Chang, Elise F. Talsma, Hongyi Cai, Shenggen Fan, Yuanying Ni, Xin Wen, Pieter van ‘t Veer, Sander Biesbroek

**Affiliations:** 1 College of Food Science and Nutritional Engineering, China Agricultural University, Beijing 100083, China; zhiyao_chang@163.com (Z.C.);; 2 Division of Human Nutrition and Health, Wageningen University & Research, 6700 AB Wageningen, The Netherlands; 3 Academy of Global Food Economics and Policy, China Agricultural University, Beijing 100083, China

**Keywords:** ultra-processed foods and drinks, sustainability, mealtimes, multilevel model, NOVA classification

## Abstract

Background: Rapid socio-economic developments confront China with a rising consumption of ultra-processed foods (UPFs) and ultra-processed drinks (UPDs). This study aims to evaluate their potential impact on diet transformation towards sustainability including nutrition, environmental sustainability, and diet-related cost. Methods: Dietary intake was assessed by 24 h recalls in 27,311 participants (age: 40.5 ± 19.7; female, 51.1%) in the China Health and Nutrition Survey 1997–2011. The nutrient quality, environmental sustainability (greenhouse gas emission (GHGE), total water use (TWU), land use (LU), and diet cost were assessed as diet-related sustainability indicators. Foods and drinks were classified according to the degree of processing based on NOVA. Two-level mixed effects models were applied to explore the secular trends of the sustainability indicators being nested within random effect (individual level). Results: UPFs and UPDs are less nutrient-dense, containing more energy, sodium, and added sugar compared to unprocessed or minimally processed foods and drinks (MPFs and MPDs). UPFs and UPDs were higher for GHGE and TWU but lower for LU. Costs of UPDs tripled those of MPDs. In the period of 1997–2011, the percentage of UPFs and UPDs per 2000 kcal increased for both sexes. The increase in UPFs and UPDs was associated with a lower nutrient quality but a higher environmental impact and diet cost. Conclusions: From 1997 to 2011, there was a significant increase in the consumption of UPFs and UPDs in China. This trend had negative impacts on both the nutrient quality and environmental impact; meanwhile, it led to increased diet costs. Policies to reduce the production and consumption of UPFs and UPDs should be reinforced by making alternatives for ultra-processed breakfast cereals, snacks, and alcoholic beverages available and acceptable. Additionally, instead of only focusing on high-UPFD consumers, attention is needed on the currently low-UPFD consumers as their consumption has been growing rapidly in the last decades.

## 1. Introduction

Diet shifts towards sustainable patterns are urgently needed for current food systems. Several studies suggest that the promotion of minimally processed and local foods is necessary, alongside the imperative to address the increasing consumption of ultra-processed foods and drinks (UFPDs) [1,2]. In itself, processing is often needed to make foods and drinks edible or palatable [3], and it is a necessity to ensure food security and food safety for a growing world population [4]. It is crucial to differentiate between impacts of processing and ingredients and additive selections that potentially make these foods unfavorable [5]. In the last two years, studies reported that UPFDs are associated with diverse health outcomes, environmental impacts, and relatively high costs [6,7,8].

To categorize foods and drinks based on their degree of processing, the most commonly used tool is the NOVA classification [9]. This classification defines UPFDs as products formulated mostly or entirely from substances derived from foods, or with little or nonwhole foods. UPFDs are often high in energy density, sugars, salt, and trans fats, as well as additives, but low in protein, vitamins, minerals, and dietary fibers [10]. UPFDs take up around 50% of total daily energy intake in high-income countries, and their consumption is increasing rapidly in low- and middle-income countries [11,12].

China is undergoing a rapid economic development associated with urbanization, which impacts food choices and availability [13]. Consumption of UPFDs nearly tripled from 62 g/d per capita in 2002 to 174 g/d per capita in 2016 based on a national-level database [14]. Most studies related to UPFDs only focused on the time trend but could not address the variation between population subgroups and the impacts of UPFD consumption from different mealtimes [13,15,16]. They also did not capture the role of individual behaviors on UPFD consumption, and few studies reported trends in ultra-processed foods (UPFs) and ultra-processed drinks (UPDs) separately.

Furthermore, individual-level analysis of food consumption according to the degree of processing is lacking, and the associations between UPFD consumption and different diet-related sustainability aspects have not yet been studied in China. Therefore, we aimed to (1) identify the characteristics (i.e., nutritional quality, environmental impact, and food cost) of foods and drinks based on the degree of processing; (2) assess the secular trend of UPF and UPD consumption according to the different mealtimes; (3) and evaluate the associations between UPFD consumption and diet-related sustainability indicators over a 14-year period.

## 2. Materials and Methods

### 2.1. Population and Dietary Data

The China Health and Nutrition Survey (CHNS) is an ongoing longitudinal cohort project [17]. CHNS collects individual-level data involving health, nutrition, and demographic factors. This study used data of round 1997, 2000, 2004, 2006, 2009, and 2011, which were derived from 12 provinces or municipalities, namely, Heilongjiang, Jiangsu, Shandong, Henan, Hubei, Hunan, Guangxi, Guizhou, Liaoning (as from 2000), Beijing, Shanghai, and Chongqing (only from 2011). In this study, participants aged 1–79 years were included, and the observations were selected separately from each round. Exclusion of the records in the dataset were based on the following criteria: lactating and pregnant female participants (n = 514), as well as those with a Z-score < −5 or >5 for energy intake (n = 285).

Dietary data were based on a combination of 24 h dietary recalls at the individual level and a food inventory taken at the household level over 3 consecutive days. Quantities of food consumption were calculated for each participant using the mean of 3 days. Mealtimes were categorized into breakfast, lunch, dinner, and snacks.

### 2.2. Classification of Foods and Drinks According to the Degree of Processing

The degree of food processing was determined by the NOVA classification system (Table S1) [9]. In the present study, foods and drinks were classified into separate categories, where all unique food and drink items reported by participants in the China Food Composition Table-2009 (FCT) were identified and systematically classified into one of the four NOVA categories [18]. The NOVA classification distinguishes four categories: (1) unprocessed or minimally processed foods, (2) processed culinary ingredients, (3) processed foods, and (4) ultra-processed foods. In addition, alcoholic drinks are not covered in the NOVA classification [9]. Therefore, beer, fruit wines, and wine were classified as processed drinks if they are produced by fermentation of unprocessed foods. Other alcoholic drinks (e.g., liquor and spirits) were classified as ultra-processed drinks if they were distilled products or industrial formulations. A research dietician cross-checked the classification and provided expert judgement.

### 2.3. Nutrient Quality

Foods and drinks from CHNS 1997–2011 were linked to Chinese FCT, in order to evaluate the daily intake of energy and the often-assessed nutrients for UPFDs (protein, dietary fiber, saturated fatty acid (SFA), sodium, and added sugar). The Nutrient-Rich Diet Score 15.3 (NRD15.3) was used as summary estimate to characterize quality of the total nutrient intakes (i.e., nutrient quality) of the whole diet [19,20]. NRD15.3 is the unweighted sum of percentage of reference daily values (RDV) for fifteen qualifying nutrients (nutrients to encourage: protein; dietary fiber; vitamins A, B1, B2, B12, C, D, E; Ca; Fe; K; I; Zn; and Se) minus the sum of percentage of maximum reference values (MRV) for three disqualifying nutrients (nutrients to limit: saturated fatty acid, added sugar, and Na) [21]. Each sub-score for qualifying and disqualifying nutrients was capped at 100 [20]. The RDV and MRV were determined based on the Recommended Nutrient Intake (RNI) values or the Tolerable Upper Intake Level (UI) values from China dietary reference intakes (DRIs), considering the different nutrient requirements by sex and age. To ensure comparability of NRD15.3 scores between regions, daily nutrient intakes were adjusted to 2000 kcal (Table S2). To calculate the intake values of components included in NRD15.3, the daily intakes of these nutrients were also calculated by linking food consumption to the FCT.

The equation of NRD15.3 Index is as follows:

(1) $$NRDX.Y=\sum_{1}^{i=X}\frac{{(Nutrient}_{i}/Energy)\times 2000}{{RDV}_{i}}\times 100-\sum_{1}^{j=Y}\frac{{(Nutrient}_{j}/Energy\times 2000)}{{MRV}_{j}}\times 100$$

where X is the number of qualifying nutrients; Y is the number of disqualifying nutrients; Nutrient i, j is the average daily intake of nutrient i or j; RDV_i_ is the reference daily value of qualifying nutrient i; and MRV_j_ is the maximum reference value of the disqualifying nutrient j. Both RDV_i_ and MRV_j_ were standardized to 2000 kcal.

### 2.4. Environmental Impact

The environmental impacts of foods were evaluated for greenhouse gas emissions (GHGE, as an air pollution indicator, measured in kg CO_2_-eq), total water use (TWU, as a water pollution indicator, measured in m^3^), and land use (LU, as a land occupation indicator, measured in m^2^). These three indicators are the closest related to the agricultural-food system and have the most representativeness, with 30% of GHGE, 70% of TWU, and 40% of LU originating from anthropogenic activities. Data on environmental impact were derived from the representative Chinese Food Life Cycle Assessment Database (CFLCAD). CFLCAD is a specialized environmental indicator database for Chinese food, which is continuously updated and has undergone validation for its effectiveness; details of the CFLCAD can be found elsewhere [22]. In brief, environmental footprints of individual diets were estimated by multiplying the quantities of food items with their footprints per unit of these three environmental impacts.

### 2.5. Diet Cost

In this study, food prices from the CHNS 2004–2011 (for the years 1997 and 2000, food price data are unavailable) food market information database at the community level were linked to dietary consumption [23]. For all food items, the lowest free market prices were used as default and substituted with the lowest retail prices wherever free market prices are missing. We assume that the lowest prices of products represented the accessibility of foods and drinks [24]. Therefore, to calculate the diet cost, the specific food price in Chinese Yuan per gram (CNY/g) was multiplied by the mean value of food consumption for each food item. The total diet cost was calculated by summing all the cost of foods consumed in the whole diets.

### 2.6. Covariates

Socio-demographic and behavior data obtained in CHNS 1997–2011 included age (in years), sex (male or female), height, weight, physical activity, smoking status, time spent on the internet, education attainment, and personal income. The Body Mass Index (BMI, kg/m^2^) was calculated by height and weight measured three times in repeat by specialized staff. The categories of physical activity were light (e.g., sedentary job, office work, watch repairers, counter salesperson), moderate (e.g., driver, electrician), and heavy (e.g., farmer, athlete, lumber worker, mason). CHNS classified education attainment as follows: no school (0 year), primary school (1–6 years), junior middle school (1–3 years), senior middle school (1–3 years), middle technical or vocational school (1–2 years), college (3–4 years in college/university), and graduate school (over 4 years in college/university). Education attainment was then divided into three categories of low (no school; primary school; junior middle school); medium (senior middle school; middle technical or vocational school), and high (college; graduate school). Urbanicity was categorized as urban and rural area according to their residential addresses at the survey time.

### 2.7. Statistical Analysis

The secular trends of socio-demographic and behavior variables were statistically tested by the Jonckheere–Terpstra method in this longitudinal study [25]. The nutritional intakes, environmental indicators, and cost for foods and drinks (per 100 g), recorded by CHNS 1997–2011, were characterized according to the degree of processing. The trends of UPF and UPD consumption according to the mealtimes were analyzed by male and female separately, in gram per 2000 kcal.

Two-level random slope and intercept mixed effect models with survey rounds (measure occasions) as level 1, nested within individual variances (level 2), were used to estimate the effect of individual UPF and UPD consumption on diet-related sustainability indicators. Model 1 was constructed with one of the NRD15.3, GHGE, TWU, LU, and diet cost transformed into Napierian logarithm as the dependent variable, with the survey rounds as independent variables (categorical). Meanwhile, the individual consumption of UPFs and UPDs in Napierian logarithm were set as a random effect (level 2). In Model 2, UPFs and UPDs in Napierian logarithm and the explanatory variables (sex, age, energy intake, BMI, activity level, smoking status, education attainment, income, and urbanicity) were added to the fixed effect.

In each model, the intra-class coefficient of correlation (ICC) was calculated as the ratio of between-individual variance to total variance of diet-related sustainability indicators [26]. The random-effects correlation coefficient was calculated to show the correlation of studied individuals between each pair of survey rounds from the random slope and intercept model [27]. To assess the goodness of fit of these models Akaike’s information criterion (AIC) was also calculated [28]. To test the stability of models, sensitivity analyses were conducted via missing value imputations. The multiple imputations (MI) by the multivariate normal distribution (MVN) method were applied to impute the missing values for meal location, height, weight, and physical activity.

The mediating effects were evaluated by the Sobel method separately for each sustainability dimension [29]. The aim of this analysis was to estimate the proportion of the total effect that is mediated by UPF and UPD consumption for the indicators NRD15.3, GHGE, TWU, LU, and diet cost and survey round.

All the five sustainability indicators and UPF and UPD consumption in these models were standardized to per 2000 kcal per person per day. All data collation and statistical analyses were performed with Stata/MP 18 (Stata Corp, College Station, TX, USA). All reported *p*-values were two-tailed, with a *p*-value < 0.05 considered statistically significant.

## 3. Results

### 3.1. Characteristics of Participants in CHNS from 1997 to 2011

The six-round longitudinal study consisted of in total 27,311 participants with 74,048 observations and a mean follow-up time of 8.1 ± 5.3 years (Table 1). In the period of 1997 to 2011, there was a 1.7-fold increase in mean energy share of UPFDs and a twofold increase in education attainment. Simultaneously, BMI, per person income, and time spent on the internet increased as well, while activity level and energy intake of participants decreased. The percentage of participants who ever smoked did not change over this period.

### 3.2. Classification of Foods and Drinks According to NOVA

**Figure 1:**
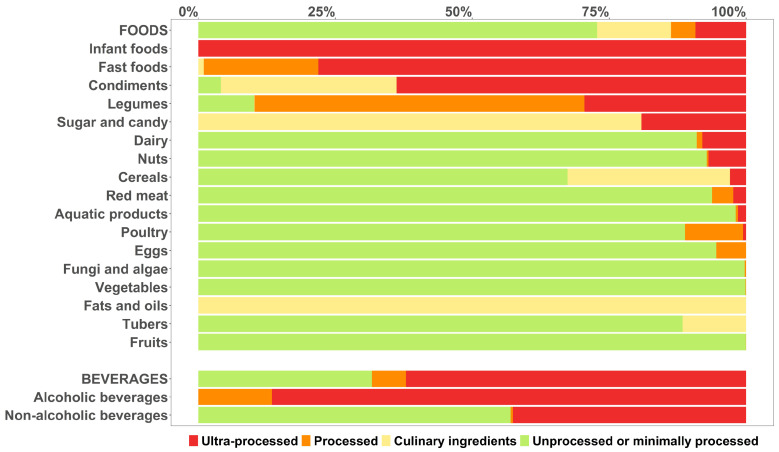
Percentage of foods and drinks according to NOVA classification for foods and drinks consumed in CHNS 2011 by food groups.

In the present study, 2435 food items in the China Food Composition Tables were categorized according to the NOVA classification. Around 30% of the foods and 75% of the drinks identified in the CHNS 2011 were categorized as ultra-processed (Figure 1). Approximately half of foods (55%) and one-fifth of drinks (19%) were classified as unprocessed or minimally processed foods and drinks (MPFDs). In the food groups ‘Infant foods’ (100%), ‘Fast foods’ (75%), ‘Condiments’ (66%), and ‘Alcoholic beverages’ (87%), the majority of foods or drinks were classified as UPFDs. The food groups ‘Fruits’ (0%), ‘Tubers’ (0%), ‘Fats and oils’ (0%), ‘Eggs’ (0%), ‘Vegetables’ (1%), ‘Fungi and algae’ (2%), ‘Poultry’ (4%), and ‘Aquatic products’ (6%) contained no or a low share of UPFDs. In addition, ‘Infant foods’, ‘Fast foods’, ‘Sugar and candy’, ‘Fats and oils’, and ‘Alcoholic beverages’ contained no MPFDs.

On average, UPFs provided around 1.6-fold more energy (211 vs. 130 kcal/100 g). As for nutrient content, UPFs contained almost 20-fold higher sodium (1153.7 vs. 59.3 mg/100) and 12-fold higher added sugar (7.2 vs. 0.6 g/100 g) compared to unprocessed or minimally processed foods (MPFs) (Table 2). UPFs contained similar amounts of SFA, and 1.3-fold more protein (7.0 vs. 5.3 g/100 g) and dietary fiber (1.5 vs. 1.1 g/100 g) compared to MPFs. UPDs had an 8.5-fold higher energy (94 vs. 11 kcal/100 g), a 2.2-fold higher sodium (12.7 vs. 5.9 mg/100 g), and a 17.5-fold higher added sugar (3.5 vs. 0.2 g/100 g) content compared to unprocessed or minimally processed drinks (MPDs). In contrast, UPDs contained only 5.3% of the dietary fiber compared to MPDs (0.01 vs. 0.19 g/100 g).

**Table 1:** Characteristics of the participants aged from 1 to 79 in the CHNS, 1997–2011 ^a^.

	All Rounds	1997	2000	2004	2006	2009	2011	*p*-Trend ^d^
Participants, n	27,311	12,416	13,211	11,552	11,091	11,151	14,627	
Females, n (%)	13,963 (51.1)	6127 (49.3)	6558 (49.6)	5871 (50.8)	5670 (51.1)	5641 (50.6)	7583 (51.8)	0.004
Age at survey time	40.5 ± 19.7	36.9 ± 19.3	36.4 ± 19.5	40.6 ± 19.5	42.4 ± 19.4	43.5 ± 19.5	43.7 ± 19.9	<0.001
BMI (kg/m^2^) ^b^	22.15 ± 6.78	20.93 ± 3.81	21.57 ± 3.93	22.13 ± 3.90	22.34 ± 4.06	22.52 ± 4.05	23.17 ± 12.63	<0.001
Smoking, n (%)	18,879 (25.5)	2990 (24.1)	2936 (22.2)	3112 (26.9)	2963 (26.7)	3067 (27.5)	3811 (26.1)	0.43
Activity level, n (%)								
Light	32,274 (43.6)	3964 (31.9)	4540 (34.4)	4888 (42.3)	4949 (44.6)	5525 (49.5)	8408 (57.5)	<0.001
Medium	19,311 (26.1)	3764 (30.3)	4087 (30.9)	3082 (26.7)	2640 (23.8)	2443 (21.9)	3295 (22.5)	<0.001
Heavy	21,510 (29.0)	4070 (32.8)	4428 (33.5)	3486 (30.2)	3436 (31.0)	3166 (28.4)	2924 (20.0)	<0.001
Education attainment								
Low	58,240 (78.7)	10,600 (85.4)	10,868 (82.3)	9174 (79.4)	8504 (76.7)	8699 (78.0)	10,395 (71.1)	<0.001
Middle	12,229 (16.5)	1603 (12.9)	1942 (14.7)	1999 (17.3)	2066 (18.6)	1929 (17.3)	2690 (18.4)	<0.001
High	3579 (4.8)	213 (1.7)	401 (3.0)	379 (3.3)	521 (4.7)	523 (4.7)	1542 (10.5)	<0.001
Income (CNY/month)	1150.7 ± 1782.3	503.1 ± 535.2	665.3 ± 801.8	823.9 ± 1024.5	1084.8 ± 1543.6	1630.8 ± 2445.9	2030.0 ± 2487.9	<0.001
Time spent on the internet (minutes)	Not applicable	Not measured	Not measured	Not measured	8.8 ± 47.9 (7.9, 9.7)	15.8 ± 62.5 (14.6, 16.9)	27.1 ± 82.7 (25.8, 28.5)	<0.001
Energy intake (kcal/day)	2059.6 ± 725.5	2202.0 ± 735.9	2203.6 ± 756.8	2108.3 ± 733.2	2046.9 ± 705.6	2011.2 ± 669.4	1816.5 ± 667.2	<0.001
UPFDs ^c^ (% energy)	6.0 ± 9.9	3.5 ± 6.4	4.1 ± 7.8	4.7 ± 8.6	6.7 ± 10.6	7.2 ± 10.6	10.4 ± 12.4	<0.001

^a^ Abbreviations: CHNS: China Health and Nutrition Survey; BMI: Body Mass Index; CHY: Chinese Yuan; UPFDs: ultra-processed foods and drinks. Continuous variables are expressed by mean and standard deviation. Categorical variables are expressed by number (percentage). The total observation was 74,048 based on 27,311 participants. ^b^ Frequency of missing values for BMI (1460 in 1997; 1551 in 2000; 915 in 2004; 939 in 2006; 682 in 2009; 538 in 2011), physical activity (618 in 1997; 156 in 2000; 96 in 2004; 66 in 2006; 17 in 2009). ^c^ UPFDs: The proportion of energy contribution of ultra-processed foods and drinks in grams per 2000 kcal per day. ^d^ *p* for trends of ordinal categorical variables were tested by the chi-squared test with Kruskal–Wallis rank method, and linear regressions were used to test the ordinal continuous variables (adjusted by gender and age).

UPFs were associated with 1.4-fold higher TWU (0.40 vs. 0.28 m^3^/100 g) and 1.4-fold higher LU (0.36 vs. 0.25 m^2^/100 g) compared to MPFs. UPFs had a similar GHGE (0.23 vs. 0.24 kg CO_2_-eq/100 g) density compared to MPFs. In addition, UPDs were associated with slightly higher GHGE (0.09 vs. 0.07 kg CO_2_-eq/100 g) but 3.1-fold lower TWU (0.16 vs. 0.50 m^3^/100 g) and 1.5-fold lower LU (0.11 vs. 0.16 m^2^/100 g) than MPDs. UPFs were 1.4-fold more expensive than MPFs (1.24 vs. 0.91 CNY/100 g), while UPDs cost three times more (2.61 vs. 0.79 CNY/100 g) compared to MPDs.

### 3.3. UPF and UPD Consumption During Mealtimes

**Figure 2:**
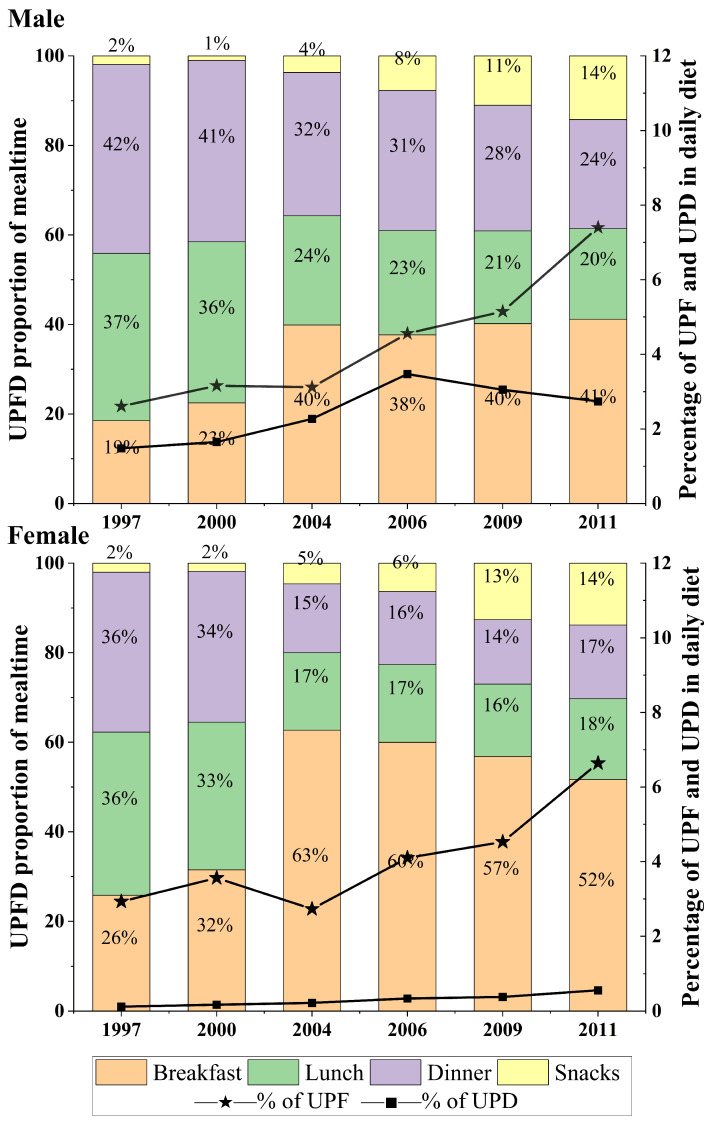
The percentage consumption of UPF and UPD (grams per 2000 kcal) in daily diets and the distribution of UPFD according to the mealtimes in the CHNS, 1997–2011. UPFD: ultra-processed food and drink.

The CHNS population consumed a daily average of 44 g (72 kcal) and 29 g (59 kcal) UPFDs in 1997, and 117 g (186 kcal) and 84 g (150 kcal) UPFDs in 2011 for male and female consumers, respectively. The percentage of UPF consumption in daily diets (g/2000 kcal) increased for both sexes between 1997 and 2011 (Figure 2). The percentage of UPD consumption (in grams per 2000 kcal) showed a similar trend (2.3 times increase) between 1997 and 2006, but slightly declined after 2006 for males. For females, there was a smooth increase in UPD consumption from 1997 to 2011. Compared to males, they only consumed 1/5 amount of UPDs, mainly due to a much lower level of alcoholic drink consumption.

The amount of UPFD consumption increased in all mealtimes over the same period. During lunch and dinner, UPFDs increased 1.7 times (24.5 to 42.7 g/2000 kcal) and 1.8 times (26.2 to 46.7 g/2000 kcal), respectively. However, the relative contribution to total daily UPFD consumption during these meals decreased by 36.9% (males) and by 35.9% (females) for lunch, and by 42.2% (males) and by 35.4% (females) for dinner. On the contrary, those contributions to total daily UPFD consumption overall increased for breakfast by 2.2 times for males and 2.0 times for females, and snacks by 7.5 times for males and 6.9 times for females (Table S3).

### 3.4. Associations of UPFD Consumption with Nutritional Quality, Environmental Impact, and Diet Cost from 1997 to 2011

The associations between sustainability indicators and UPFs or UPDs were examined by two-level mixed effect models (Table 3). Over the 14-year period, the NRD15.3 score increased by around 16%, which was similar to TWU (17%), while GHGE (31%) and LU (22%) increased more. Diet cost increased more than twofold over only 7 years. Thus, diet cost, GHGE, and LU increased at a much faster rate than diet quality (NRD15.3) and TWU. Given the trend in UPF consumption, UPF consumption was not associated with diet quality (NRD15.3), but doubling consumption of UPFs increased environmental impacts in GHGE (1.4%), TWU (2.2%), and LU (2.9%), as well as diet cost (2.9%). On the other hand, doubling UPD consumption was associated with decreased NRD15.3 (−1.0%) but positively associated with GHGE (5.6%), TWU (4.9%), and LU (3.3%), and most strongly in diet cost (19.9%). UPDs had fourfold more, twofold more, and sevenfold less effects on GHGE, TWU, and diet cost. There were only small effects on NRD15.3, GHGE, and diet cost mediated by UPFDs, which amounted to −2.7%, 2.8%, and 3.0%, respectively. Conversely, the proportion of total mediating effects for TWU and LU were relatively larger (27.9% and 11.5%, respectively) (Table S5).

For the demographic variables, female sex (coefficient = 0.047) and older age were associated with higher diet quality (NRD15.3). Diet quality was positively associated with individual income (coefficient = 0.005, per 1000 CNY/month) and education attainment (coefficient = 0.028, medium level to low level) as well. In contrast, the individuals who had ever smoked (coefficient = −0.021), had higher energy intake (coefficient = −0.004, per 100 kcal), and had lower activity levels also tended to have lower NRD15.3 scores. People living in urban areas had around 5.1% higher NRD15.3 scores, while they would spend 11.2% more on their diets, and their diets tended towards higher environmental impacts. The three environmental indicators were positively associated with education attainment (5.45%, 6.17%, and 7.47% higher for GHGE, TWU, and LU, respectively, in the high education attainment group), smoking, and individual income. Conversely, the individuals who had more active lifestyles and spent less time online tended to have lower GHGE, TWU, and LU in daily diets. The trend in diet cost from 2004 to 2011 was negatively associated with activity level and energy intake (coefficient = −0.007, per 100 kcal). In particular, participants with heavy physical activity levels had 16.1% more expenditure on diets than those with light physical activity levels, while diet cost was not associated with BMI. In addition, diet cost was positively associated with individual income and education attainment. Overall, around 42% and 73% of the variances in GHGE and diet cost could be attributed to variation between individuals, which means that a large portion of the association is encapsulated in inherent individual dietary consumption behaviors that cannot be attributed to the explanatory variables. After imputation, the coefficients and significance of all models in sensitivity analyses showed the same trends of sustainability indicators, demonstrating that the models have high validity, and the conclusions are robust and reliable (Supplementary Table S6).

## 4. Discussion

### 4.1. Summary of the Main Results

Based on the NOVA classification, we found that 30% of the foods and 75% of the drinks consumed in this study were UPFDs. On average, UPFs and UPDs contained, respectively, 1.6-fold and 8.5-fold higher energy and 12-fold and 17.5-fold higher added sugar per 100 g compared to MPFs and MPDs. They had similar density (per 100 g) for GHGE, but less LU compared to MPFs and MPDs, and UPDs cost three times more compared to MPDs. From 1997 to 2011, the proportion of UPFs and UPDs in diets increased for both males (2.5 times) and females (2.3 times), but UPDs remained 80% lower in females compared with males. The relative contribution to total daily UPFD consumption during mealtimes increased two times for breakfast and seven times for snacks, and therefore decreased in lunch (−36%) and dinner (−38%). All sustainability indicators increased over the study period and were positively associated with the increased consumption of UPFDs. Furthermore, diet cost had risen the most among all indicators, which was correspondingly explained the most by the between-individual heterogeneities (~73%). In addition, UPFD consumption had an undeniable mediating effect on the trends of TWU and LU with 27.9% and 11.5% of the total effect, respectively.

### 4.2. Foods and Drinks Categorized According to NOVA Classification

This is the first study to evaluate multiple sustainability dimensions of UPFDs in China according to the NOVA classification. The NOVA classification system has been widely applied to conduct category analyses for UPFDs in China [13,30]. Compared with high-income countries, food groups like candy, fats and oils, condiments, and alcoholic beverages in China were similarly identified with containing higher proportion of UPFs or UPDs [31]. Instead, food groups, such as cereals (−70%), red meat (−35%), tubers (−44%), and nuts (−13%) were less processed in China [15]. In contrast, legumes contained a relatively higher percentage of UPFs, which were 25% less processed in high-income countries. This might be explained by the different consumption patterns between high-income countries and China. For instance, food items like ultra-processed bread and breakfast cereals are often consumed as staple foods in the Netherlands and America, whereas in China, steamed rice and steamed sweet potatoes are the most commonly consumed ones [31,32]. Furthermore, according to the NOVA classification, all infant foods are categorized as ultra-processed. However, this does not mean that these foods are necessarily unhealthy [33]. With a series of standards and regulations in China, the stunting rates gradually decreased among infants [34,35], indicating that infants do not face high health risks. Additionally, tofu, as a traditional Chinese food, is consumed at much higher rates within the legume category compared to other countries and regions [36].

In terms of comparing the characteristics of UPFDs and MPFDs in daily diets, our results are in line with previous studies [31,37]. The lower nutrient quality observed in our study due to increased consumption of UPFDs is associated with their low-fiber, high-sugar, and high-sodium characteristics. This finding is consistent with previous studies indicating that foods rich in energy, sodium, and low in dietary fiber are linked to significant declines in health status [38]. Our study examined the difference between MPFDs and UPFDs regarding GHGE, TWU, LU, and cost, finding a positive association between UPD consumption and GHGE, albeit with no evidence linking high UPF consumption to increased GHGE and LU in current Chinese diets. This might reflect the fact that in China, the proportion of ultra-processed animal-based foods, such as red meat, is around 50% lower compared to high-income countries like the US [39,40].

### 4.3. Trends of UPFD Consumption According to Mealtimes

Within the Healthy China program, the concept of healthy eating has been continuously promoted and disseminated, and it has received increasing attention [41]. However, similar to most countries around the world, the proportion of UPFD consumption is still rising with the increased ongoing socio-economic development [42]. In addition, we found that the consumption of UPFDs among males increased faster compared to females, especially for UPD consumption, wherein males currently consumed an average 22-fold more alcoholic drinks per day than females, and the trend continues to widen. This reflects the typical Chinese drinking culture in relation to the work banquets, where male workers may be encouraged to have more alcohol to demonstrate deeper emotional connections [43].

Based on energy-standardized dietary intake, shared proportion of UPFDs in breakfast nearly doubled from 1997 to 2011. This may be influenced by the increasing habit of skipping traditional breakfast and having convenient package breakfast, such as ready-to-eat oats and processed grains. This might be influenced by the Breakfast Cereal Fortification Strategy initiated in 2000, as well as the pilot projects for processed breakfast cereals launched in 2002 [44,45,46,47]. Similarly, the proportion of UPFDs in snacks also increased by 12% over this period, and both the increased amount and industrial formulation of snacks would contribute to this phenomenon [48]. Although the shared proportion of UPFDs in dinners and lunches has gradually decreased within total mealtime, the continued rise in the amount of UPFD consumption warrants attention. As mentioned earlier, there is a tendency for individuals to opt for convenient UPFDs over traditionally cooked meals in terms of breakfast and snacks. However, the increasing affordability tends to make dining out more common, and consequently it slows the growth of UPFD proportions in lunches and dinners [49].

### 4.4. Trajectory of Sustainability Indicators (1997–2011) Mediated by UPFD Consumption

This study also firstly investigated the trend of diet-related nutritional quality, environmental impacts, and cost mediated by UPFD consumption in a Chinese population. In our study, from 1997 to 2011, all estimated indicators increased, albeit with a relatively small increase in diet quality compared to the relative increase in the other indicators, due to the hindrance effect mediated by the simultaneous increased UPFD consumption, i.e., rapid growth in UPFD consumption accelerated the diet-related environmental deterioration and food expenditure. This is also in accordance with the finding of characteristics of UPFDs in relation to environmental impacts in previous studies [31], especially UPFDs containing nearly 1.5-fold higher LU per unit. The cost of UPFDs in the rising dietary expenditure was further increasing, which resulted in an almost doubling of the total diet cost during a 7-year period. Headey and Alderman highlighted that fat-, salt-, and sugar-rich foods have a 5–10 times higher calorie prices in low- and middle-income countries compared to high-income countries [50]. This also implies that they are less affordable for people with the lower education and income, i.e., the subgroup in the rural areas. This aligns with the characteristics of UPFDs of high price but low nutrition density; therefore, contrary to high-income countries, the increasing consumption of UPFDs indirectly accelerated the diet cost in China. This indicates that emphasis should be placed on both variations in food groups (i.e., dietary patterns) and levels of food processing during dietary transitions, as neglecting these factors may result in disregarding the detrimental impacts of food processing on nutrition, environmental sustainability, and dietary costs, for instance, regulating food industries to encourage the shift towards environmentally friendly production and processing.

Additionally, even though UPFDs have been continuously increasing during the studied period, the overall dietary energy intake has decreased. This is mainly because more than 50% of the energy in the study population comes from cereals, and as their dietary patterns have changed over time, the consumption of cereals has significantly decreased. This resulted in reduced energy intake on average.

### 4.5. The Heterogeneity of Individual Dietary Habits

When adjusting the trends in sustainability indicators for the simultaneous changes in socio-economic factors, we found that smoking, lower physical activity level, and more time spent on the internet were all positively associated with increasing diet-related environmental impacts and cost, but lower nutrient quality, which appeared to be mediated by increased consumption of UPFDs. This was also approved by previous publications that increased exposure to the internet and media provides more opportunities for accessing advertisements related to UPFDs [51,52]. As highly educated and high-income people were mostly living in urban areas, the growth rate of diet cost is approximately 11.3% higher in urban areas than in rural areas. This is primarily due to the rapid reduction of arable land around cities and the increasing reliance on imported agricultural products [53,54]. This corresponds to results of previous studies that reported a strong correlation between unhealthy lifestyles and high consumption of UPFDs [55].

On the other hand, the effects of unmeasured behaviors internalized in individuals are represented by the ICC with 20–40% variance in nutrient quality and diet-related environmental impact. This revealed that such behaviors like eating habits or food preferences had a vital influence on bad food choices like UPFs and UPDs, leading to reduced nutrient quality and increased environmental footprints [12]. The inherent differences in diet cost among individual consumers are the most significant, with over two-thirds of the difference in food expenditure reflected by individuals’ willing on UPFDs purchase. Therefore, when regulating diet transitions at the individual level, policies focusing on food prices might be better to reduce healthy food prices such as with fresh vegetables and fruit. Furthermore, the random-effects correlation coefficients and variance of interaction were all negative values throughout the multilevel models, which represents the estimated individual correlation between each pair of survey rounds [56,57]. For example, GHGE had a coefficient of −0.157 in this study, which means that consumers with a higher starting point (above-average GHGE per 2000 kcal in 1997) tend to have a lower growth rate in future UPFD consumption, and vice versa.

Obviously, the inherent dietary habits are inevitably influenced by their food environment [58]. As is commonly acknowledged, alongside the rapid socio-economic development, the work pace of individuals, notably in metropolitan cities, has intensified. Traditional domestic culinary practices prove inadequate in accommodating the time constraints imposed by contemporary lifestyles, and especially for high-educated populations, they tend to have less time for housework, including cooking [59]. In response to the need for time and labor conservation, convenience retailers have swiftly emerged in densely populated regions, markedly enhancing the accessibility and visibility of UPFDs. For instance, in most supermarkets, because businesses can profit more from highly processed foods [60], they are placed in more accessible locations and occupy a significant proportion for convenient purchase [61,62]. This is also related to certain individual lifestyle habits, reflecting the broader food environment of society. More people are abandoning traditional cooking due to the modern busy lifestyle [63], and UPFDs offer a simpler and quicker option. Especially for high-income people, UPFDs can be a convenient choice after a busy and exhausting workday. Therefore, in dietary transitions, a key focus should be to guarantee that consumers can easily experience and enjoy the healthy and sustainable benefits of MPFDs amid their fast-paced lifestyles.

### 4.6. Rethinking the Environmental Impact of Ultra-Processed Foods and Drinks

The categorization of foods according to NOVA for health-related outcomes is widely discussed and frequently used in food education [64]. In light of current diet-related non-communicable diseases and climate change, alongside increasing disparities in economic status, integrated measures that address nutritional quality, environmental impact, and diet cost holistically are preferred over singular approaches like NOVA-based dietary health recommendations. Effective dietary advice should ideally harmonize nutritional quality, environmental impact, and cost considerations for both foods and beverages to facilitate a sustainable transition. Although NOVA was originally designed to gauge the extent of food processing, its applicability in assessing health, environmental, and cost impacts appears limited. Our findings reveal inconclusive and divergent outcomes when applying the NOVA framework to evaluate environmental impact between ultra-processed and minimally processed food diets, which also underscores the uncertainty and heterogeneity in the environmental impact of UPFDs [65]. Thus, there is skepticism regarding the necessity of food classification based on NOVA as a viable methodology for environmental assessments or as a foundational element for sustainability-related food policy. An alternative method to NOVA should be able to meticulously quantify the different resources consumed in food processing, and under this circumstance, the differences in environmental impacts can become more apparent, allowing for a clear distinction and concrete changes in environmental sustainability.

However, the undeniable increase in total water and land footprints due to the rising consumption of UPFDs in China confirms the need of implementing policies such as substituting high-water-footprint foods in diets, like ultra-processed cereal [66], to effectively alleviate ecological pressures from agricultural systems. Therefore, it is crucial to investigate the connection between (ultra-)processed foods and their environmental and economic dimensions across different contexts.

### 4.7. Policy Implications

Our research carries significant policy implications in relation to public health and diet-related environmental impact. Most importantly, it is imperative to curb the rapidly increasing consumption of UPFDs in China. In particular, based on the results of noticeable heterogeneity of individual UPFD consumption behaviors, the low-UPFD groups are supposed to be restricted from gradually catching up UPFD consumption as soon as possible [67]. Secondly, China currently lacks concrete policies to respond to the increasing production and consumption of UPFDs [68], in addition to proposing and promoting national-level guidelines for public nutrition and health. In order to address the aforementioned substantial shares of ultra-processed breakfast cereals and ultra-processed beverages consumed during breakfast and snack times, short-term implementation measures should be designated promptly. To address the decline in diet affordability, it is advisable to implement policies that promote local food consumption, such as restricting imports or providing subsidies for local agricultural production [54,69], in order to counteract the rise in ultra-processed food consumption and prices.

Moreover, policymakers could employ fiscal measures such as a ‘UPFDs tax’ to make healthier options more affordable, educational campaigns to raise awareness about not only the health but also the environmental impacts of UPFDs [70], and regulatory measures to encourage food industry reform towards more sustainable production practices. Apparently, these aforementioned policies are feasible in a Chinese context. The Chinese government has increasingly prioritized environmental sustainability and supports environmental-friendly food processing, as evidenced by its commitment to carbon neutrality by 2060 [71]. This creates a favorable environment for implementing fiscal measures, such as subsidies for sustainable alternatives or taxes on UPFD products, which could gain traction among policymakers. However, although the feasibility is high to implement those measures, there are still several challenges that will be encountered. The economic impact on industries and cultural acceptance would be the top two concerns. Industry sectors might lobby against fiscal measures due to concerns about increased costs and potential job losses [72]. There may be cultural preferences for convenience products. Overcoming these entrenched consumer habits could lead to resistance against educational campaigns that promote behavioral change [73]. Both are creating a significant barrier to policy implementation. Meanwhile, the inconsistent environmental impacts of UPFDs across various research and classification contexts highlight the necessity for developing generally applicable food classification tools according to their degree of processing that encompass environmental and economic considerations. These approaches will provide more rigorous theoretical frameworks and practicality for future categorization and assessments of UPFDs under the sustainable dietary transitions.

### 4.8. Limitations

Firstly, NOVA classification, applied in the current study, is currently the only standardized and commonly used tool to classify foods and drinks according to their degree of processing, crucial for research in nutrition and public health [31]. It should be noted that nutrient quality and environmental impacts of foods and drinks prepared at home could be worse than that of ultra-processed foods [10]. However, categorizing foods and drinks prepared at home using NOVA faces challenges due to insufficient standardization [74], leading to potential confusion and debate within these categories. In our study, detailed data on food preparation methods allowed us to systematically apply NOVA classification to homemade foods and drinks with minimal inconsistency or subjective judgment. For example, for fried chicken prepared at home, we also classified them into the processed or ultra-processed groups of NOVA classification based on their (deep) frying cooking methods and (industrial) ingredients, thereby reducing the underestimation of UPFD-related health outcomes. Secondly, takeaway meals and dining out often involve greater exposure to UPFDs, making it relevant to be considered in UPFDs’ consumption [15]. A portion of takeaway meals and dining out is not recorded in the CHNS dataset, posing a risk of underestimating the proportion of UPFDs in this study. However, as of 2011, less than 2.5% of food consumption came from takeaway meals and dining out. Relevant studies [75,76] have also reported that these dietary behaviors are related to lifestyle characteristics, so the impact from the missing value can partly be explained by covariates we included in our models. In addition, the sensitivity analyses (Supplementary Table S6) showed similar trends of all sustainability indicators and their association with covariates. Therefore, it will not impact significantly on the findings in the present study. Thirdly, our analyses are based on dietary data from the CHNS 1997–2011, as more recent data were not publicly available. Nevertheless, the aim of our study was to exploit the trajectory of UPFD consumption and its impact on diet-related sustainability in China to identify opportunities for change, rather than providing a representation of current dietary patterns for China as a whole. Moreover, based on the national-level data from National Bureau of Statistics, the time trends in overall dietary pattern changes have been relatively small [77,78]. Despite a slight increase in the proportion of animal-based foods from 9.1% to 10.9% over the past decade, the Chinese population predominantly adheres to a plant-based diet in 2021, with cereals at 35% and vegetables and fungi at 26.6%. Notably, red meat (8.0%) and poultry (2.9%) maintain low proportions, similar to the CHNS 2011 (Supplementary Table S7). Consequently, utilizing the 2011 CHNS data is unlikely to significantly impact the generalizability of our conclusions regarding the associations between health and environmental sustainability in Chinese diets. Therefore, it is unlikely that using data from the CHNS 1997–2011 seriously hampers that aspect of our conclusions.

## 5. Conclusions

In conclusion, the rapid increase in UPFs and UPDs contributed to reduced diet quality and increased diet-related environmental impacts and cost in China. This decline in nutritional quality aligns with global trends where UPFDs contribute substantially to daily energy intake but offer limited essential nutrients. Based on the current consumption and time trends and policies, the Chinese government, to reduce UPF and UPD consumption, should especially focus on alternatives for processed breakfast cereals, snack products, and alcoholic beverages. Strategies could include fiscal measures to make healthier options more affordable, educational campaigns to raise awareness about the health and environmental impacts of UPFDs, and regulatory measures to encourage food industry reform towards more sustainable production practices. In addition, instead of only focusing on current high-UPFD consumers to prevent their imminent health risk, attention is needed on a large group of current Chinese low-UPFD consumers since their consumption has been changing rapidly in the last years.

## Figures and Tables

**Table 2:** The average nutrient quality, environmental impact, and cost of foods and drinks per 100 g by degree of processing in the CHNS ^a^.

	n	Energy (kcal)	Protein (g)	Dietary Fiber (g)	SFA (g)	Added Sugar (g)	Sodium (mg)	GHGE (kg CO_2_-eq)	TWU (m^3^)	LU (m^2^)	Food Cost (CNY/day) ^b^
All	2435	167.9 ± 0.5	5.71 ± 0.02	1.15 ± 0.01	6.08 ± 0.51	1.23 ± 0.02	338.49 ± 3.85	0.23 ± 0.01	0.31 ± 0.01	0.27 ± 0.01	1.01 ± 0.02
Foods	2224										
Unprocessed and minimally processed (MPFs)	1263	130.0 ± 0.6	5.33 ± 0.02	1.13 ± 0.01	0.28 ± 0.01	0.57 ± 0.01	59.29 ± 0.91	0.23 ± 0.01	0.28 ± 0.01	0.25 ± 0.01	0.91 ± 0.02
Processed culinary ingredients	158	368.0 ± 2.3	6.20 ± 0.07	1.27 ± 0.02	2.05 ± 0.01	1.31 ± 0.05	1486.28 ± 15.36	0.17 ± 0.01	0.25 ± 0.01	0.26 ± 0.01	1.39 ± 0.04
Processed	193	164.0 ± 2.4	11.63 ± 0.13	1.18 ± 0.04	1.55 ± 0.02	1.31 ± 0.17	189.52 ± 2.18	0.23 ± 0.01	0.59 ± 0.01	0.24 ± 0.01	1.11 ± 0.02
Ultra-processed (UPFs)	610	210.6 ± 2.4	7.04 ± 0.06	1.48 ± 0.2	0.28 ± 0.01	7.22 ± 0.11	1153.65 ± 4.02	0.24 ± 0.01	0.40 ± 0.01	0.36 ± 0.01	1.24 ± 0.02
Drinks	211										
Unprocessed or minimally processed (MPDs)	35	10.8 ± 1.5	0.39 ± 0.0.03	0.19 ± 10.01	0	0.23 ± 0.07	5.91 ± 0.79	0.07 ± 0.01	0.50 ± 0.13	0.16 ± 0.03	0.79 ± 0.15
Processed	14	71.6 ± 7.6	1.27 ± 0.09	0	0	12.51 ± 2.86	3.21 ± 0.65	0.08 ± 0.01	0.05 ± 0.01	0.03 ± 0.01	2.87 ± 0.38
Ultra-processed (UPDs)	162	93.9 ± 3.8	0.40 ± 0.03	0.01 ± 0.01	0	3.51 ± 0.21	12.73 ± 0.77	0.09 ± 0.01	0.16 ± 0.01	0.11 ± 0.01	2.61 ± 0.15

^a^ Abbreviations: SFA: saturated fatty acid; GHGE: greenhouse gas emission; TWU: total water use; LU: land use; CNY: Chinese Yuan. All values are presented as mean ± standard deviation. For nutrient density, see Table S4. ^b^ Food costs were only assessed from 2004 to 2011.

**Table 3:** Coefficients of two-level mixed effect models for nutrient quality (NRD15.3), diet-related environmental impacts, and diet cost among participants aged 1–79 years, CHNS 1997–2011 ^a^.

	NRD15.3		GHGE log_e_ (kg CO_2_-eq/2000 kcal)		TWU log_e_ (m^3^/2000 kcal)		LU log_e_ (m^2^/2000 kcal)		Diet Cost log_e_ (CNY/d·2000 kcal)	
**Effects**	**Model 1**	**Model 2**	**Model 1**	**Model 2**	**Model 1**	**Model 2**	**Model 1**	**Model 2**	**Model 1**	**Model 2**
*Fixed effects (level 1)*										
Intercept	6.767 ***	6.885 ***	0.762 ***	1.040 ***	1.216 ***	1.369 ***	0.963 ***	1.262 ***	1.664 ***	1.127 ***
Survey round (ref. = 1997) ^b^										
2000	−0.014	−0.003	−0.009	0.007	−0.022	−0.014	−0.037 *	−0.027	Not measured	
2004	0.051 ***	0.041 ***	0.134 ***	0.098 ***	−0.032	−0.071 ***	0.054 **	0.018	As ref.	
2006	0.079 ***	0.062 ***	0.188 ***	0.125 ***	0.031	−0.034	0.093 ***	0.028	0.083 ***	0.066 **
2009	0.099 ***	0.074 ***	0.231 ***	0.139 ***	0.074 ***	−0.024	0.130 ***	0.042 *	0.484 ***	0.438 ***
2011	0.140 ***	0.094 ***	0.278 ***	0.147 ***	0.142 ***	0.009	0.198 ***	0.068 ***	0.967 ***	0.931 ***
UPFs (log_e_ (g/2000 kcal))		0.002		0.011 **		0.020 ***		0.025 ***		0.026 ***
UPDs (log_e_ (g/2000 kcal))		−0.010 ***		0.055 ***		0.049 ***		0.033 ***		0.182 ***
Age (per 10 years)		0.005 *		−0.028 ***		−0.019 ***		−0.025 ***		−0.005
Sex (ref. = male)		0.047 ***		0.011		0.031		−0.006		0.030
Lifestyle factors										
Energy intake (per 100 kcal)		−0.004 ***		−0.009 ***		−0.009 ***		−0.009 ***		−0.007 ***
BMI (kg/m^2^)		0.002 **		0.003 *		0.003 *		0.004 **		0.001
Activity level (ref. = Light)										
Moderate		−0.006		−0.025		−0.005		−0.023		−0.033
Heavy		−0.025 **		−0.131 ***		−0.093 ***		−0.117 ***		−0.094 ***
Ever smoked (ref. = never smoke)		−0.021 **		0.009		0.016		0.019		0.016
Time spent on the internet (per 10 min)		0.001 *		0.003 ***		0.004 ***		0.004 ***		0.003 **
Socio-economic factors										
Education attainment (ref. = Low)										
Medium		0.028 ***		0.043 **		0.054 ***		0.044 **		0.060 ***
High		0.015		0.053 **		0.060 **		0.072 ***		0.058 *
Income (per 1000 CNY/month, inflated to 2011)		0.005 ***		0.007 ***		0.008 ***		0.006 **		0.007 **
Urbanicity (ref. = urban)		−0.052 ***		−0.132 ***		−0.099 ***		−0.138 ***		−0.119 ***
*Random effects (level 2)*										
Variance of slope ^c^	0.001	0.001	0.004	0.001	0.006	0.001	0.007	0.003	0.044	0.016
Variance of intercept	0.003	0.006	0.052	0.044	0.078	0.025	0.099	0.027	0.899	0.228
Variance of interaction	−0.001	−0.001	−0.001	−0.001	0.001	−0.001	0.001	−0.001	0.003	−0.001
Variance of residual	0.022	0.020	0.065	0.062	0.067	0.067	0.071	0.071	0.090	0.084
Random-effects correlation coefficient	−0.996	−0.994	−0.037	−0.153	−0.690	−0.557	−0.651	−0.175	−0.666	−0.043
ICC ^d^	0.120	0.231	0.444	0.415	0.540	0.272	0.582	0.276	0.909	0.731
AIC	3673.7	3287.1	3357.4	1457.2	3105.2	1403.5	3585.3	1689.3	4031.9	1862.3

^a^ Abbreviations: NRD15.3: nutrient-rich diet index 15.3; GHGE: greenhouse gas emission; TWU: total water use; LU: land use; CNY: Chinese Yuan; UPFs: ultra-processed foods; UPDs: ultra-processed drinks; ICC: inter-class correlation coefficient; AIC: Akaike information criterion. Level 1 represents the within-individual variations, which was assessed via the measure occasion; Level 2 represents the between-individual variations; NRD15.3, GHGE, LU, TWU, diet cost, UPD, and UPF were all transformed in Napierian logarithm form, and the NRD15.3 was calculated based on energy-standardized nutrient intake (2000 kcal per day). Model 1: included measurements and individual variables; Model 2: added the percentage of ultra-processed food and drink consumption as the mediating variable, and covariates to Model 1. *** indicates *p*-value < 0.001; ** indicates *p*-value < 0.01; * indicates *p*-value < 0.05. ^b^ For the cost of diet, the survey year is referenced to 2004. ^c^ The random slope is combined by rooting the interaction terms of ln (UPFs) and ln (UPDs). ^d^ The ICC is the proportion of the total variance that can be attributed to different trends between individuals.

## Data Availability

Data are available in a public, open access repository. The datasets analyzed in the current study are available online (https://www.cpc.unc.edu/projects/china/data/datasets/data_downloads/longitudinal, accessed on 20 December 2024).

## Supplementary Materials

The following supporting information can be downloaded at https://www.mdpi.com/article/10.3390/nu17020334/s1. Table S1: Processed foods categories based on NOVA classification for the China composition table. Table S2: Components of the Nutrient-Rich Diet 15.3 Index. Table S3: The percentage of ultra-processed food and drink consumption from 1997 to 2011. Table S4: The average nutrient quality per 1000 kcal of foods and drinks by degree of processing in the CHNS. Table S5: Mediating effect of UPFD consumption on the association between NRD15.3, GHGE, TWU, LU, diet cost, and survey round. Table S6: Coefficients of two-level mixed effect models for nutrient quality (NRD15.3), diet-related environmental impacts, and diet cost in sensitive analysis, CHNS 1997–2011. Table S7: Proportion of Food consumption (%) in China from National Bureau of Statistics, 2013–2021 and in CHNS 2011.

## Abbreviations

The following abbreviations are used in this manuscript:

UPFDs	Ultra-processed foods and drinks
UPFs	Ultra-processed foods
UPDs	Ultra-processed drinks
CHNS	China Health and Nutrition Survey
FCT	China food composition table
NRD15.3	Nutrient-Rich Diet Score 15.3
RDV	Reference daily values
MRV	Maximum reference values
RNI	Recommended Nutrient Intake
UI	Tolerable Upper Intake Level
DRIs	Dietary reference intakes
GHGE	Greenhouse gas emissions
TWU	Total water use
LU	Land use
CFLCAD	Chinese Food Life Cycle Assessment Database
CNY	Chinese Yuan
BMI	Body Mass Index
ICC	Intra-class coefficient of correlation
AIC	Akaike’s information criterion
MPFDs	Unprocessed or minimally processed foods and drinks
