# Supplementary material for: Trajectories of Nutritional Quality, Diet-Related Environmental Impact, and Diet Cost in China: How Much Does Ultra-Processed Food and Drink Consumption Matter?
Source: Nutrients. 2025 Jan 17;17(2):334. doi: 10.3390/nu17020334 (PMC11768330; doi:10.3390/nu17020334)
Supplement: Supplementary file 1 [file nutrients-17-00334-s001.zip › nutrients-3430090-supplementary.pdf]

Supplementary materials for "Trajectories of nutritional quality, diet-related environmental impact, and diet cost in China: How much do ultra-processed foods and drinks consumption matter?"

This supplementary file mainly includes all supplementary tables that were cited in the manuscript.

Table S1. Processed foods categories based on NOVA classification for China composition table

Food group (FOODCODE)	Unprocessed or minimally processed foods and drinks	Processed culinary ingredients	Processed foods and drinks	Ultra-processed foods and drinks
**Cereals (1)**	Conservation method is fresh, vacuum, dried, unknown	Starches and flours, all kinds of milled grains, uncooked noodles	Conservation method is canned	Fried cereals, cereal products by adding salt, sugar, or oils, fortified cereal products
**Tubers (2)**	Conservation method is fresh, vacuum, frozen, dried, home-made, unknown	Starches, all kinds of milled tubers		
**Legumes (3)**	Conservation method is unknown, frozen, dried, fresh, vacuum, canned, or jarred.	All kinds of milled beans	Conservation method is marinated, canned, or jarred with added sugar, salt or fat, or unknown, tofu (tofu is a traditional Chinese food, and not covered in NOVA, while it is usually homemade in China and added less additives)	Soybean milk with added sugar, salt, or malt, puffed beans, fried beans
**Vegetables (4)**	Conservation method is unknown, frozen, dried, fresh, vacuum, canned, fermented, or jarred.		Conservation method is marinated, canned, or jarred with added sugar, salt, or fat, or unknown	
**Fungi and algae (5)**	Conservation method is unknown, frozen, dried, fresh, vacuum, canned, fermented, or jarred.		Conservation method is marinated, canned, or jarred with added sugar, salt, or fat, or unknown	
**Fruits (6)**	Conservation method is not known, frozen, dried, fresh, heat treated, canned, jarred or medium is in water.		Conservation method is marinated, confit, jarred or canned with added sugar, salt or fat, and	Industrially prepared (only those foods that have lost the original sensory characteristics and a significant amount of nutrients,

Food group (FOODCODE)	Unprocessed or minimally processed foods and drinks	Processed culinary ingredients	Processed foods and drinks	Ultra-processed foods and drinks
			medium is in syrup, juice or unknown	and that have added industrial food additives)
**Nuts and seeds (7)**	Unsalted or if salt content is not specified		Salted, sugared	Industrial formulations (added ingredients other than seasonings, such as starch coatings used for frying, or industrial food additives that improve sensory characteristics)
**Meat, meat products and substitutes (8)**	If conservation method is fresh, not specified, frozen or vacuum.		If conservation method is canned or jarred, dried, salted, smoked, marinated, processed meat may contain additives used to preserve their original properties or to resist microbial contamination, such as ham, bacon, pastrami and similar.	Meat including industrial formulations, all kinds of meat substitutes, meats for which processes include hydrogenation, hydrolysis, extruding, moulding, reshaping, pre-processing by frying, baking; meat containing additives not used to preserve or to resist microbial contamination; pre-prepared meat and other reconstituted meat, such as nuggets, sticks, sausages, burgers, hot dogs.
**Poultry (9)**	If conservation method is fresh, not specified, frozen or vacuum.		If conservation method is canned or jarred, dried, salted, smoked, marinated, processed poultry may contain additives used to preserve their original properties or to resist microbial contamination, such as roast chicken.	Poultry including industrial formulations, pre-prepared poultry, and other reconstituted poultry, such as nuggets, sticks, sausages, burgers, fried chicken.
**Dairy (10)**	Not sweetened, not specified if sweetened;		If added sugar, salt, or fat, cheese, all kinds of dairy creams, and their products	If sweetened, additives, colours, emulsifiers added, spreadable cheese, all kinds of non-dairy creams, and their products

Food group (FOODCODE)	Unprocessed or minimally processed foods and drinks	Processed culinary ingredients	Processed foods and drinks	Ultra-processed foods and drinks
**Eggs and egg products (11)**	Not salted, not canned		Conservation method is marinated, jarred, or canned with salt	
**Fish, crustaceans, mollusc, amphibians, and reptiles (12)**	If conservation method is fresh, frozen, unspecified, vacuum		If conservation method is canned or jarred, or in oil, marinated, smoked/salted fish	Fish in crumbs, fried fish, fish including industrial formulations, pre-prepared fish, and other reconstituted fish, such as fish nugget, fish ball
**Infant formulas (13)**				All foods are categorized as UPF
**Fast food and snacks (14)**			If homemade (database in the present study has detail description of cooking or preparing methods), not fried, not include reconstituted meat, not with UPF ingredients	Industrial formulations, fried, or reconstituted meat, cakes, breads, buns, biscuits, crackers, breakfast cereals, desserts, pizza, pasta dishes, ice cream, jelly, chips, fries, dim sum, burgers, sandwich, instant foods, dumplings, puddings,
**Sugar, honey, syrup, preserves (17)**		Sugar, honey		All foods are categorized as UPF, except for the processed culinary ingredients.
**Fats and oils (18)**		Vegetable oils, butter, animal fats		Margarines
**Condiments, spices, sauces, jam, and yeast (19)**	Fresh	Vinegars	If homemade sauces without ingredients being disaggregated	If consistence is powder or concentrate. Industrial prepared sauces or sauces with unknown preparation method.
**Drinks**				
**Non-alcoholic beverages (15)**	Water (bottled), tea, coffee, homemade 100% fruit or vegetable juices		Fruit or vegetable juices with added sugar	Industrial formulations, carbonated/soft/isotonic drinks apart from condiments added
**Alcoholic beverages (16)**			Homemade (non-industrialized winery) wine, ciders, fruit	Spirits, whiskey, brandy, vodka, liqueurs, mixed punches, cocktails, and

Food group (FOODCODE)	Unprocessed or minimally processed foods and drinks	Processed culinary ingredients	Processed foods and drinks	Ultra-processed foods and drinks
			wines, sherry, beer	all other industrial wines, beers, and fruit wines

Table S2. Components of Nutrient-Rich Diets 15.3 Index

NRD component	RDA and MRV		Standards from DRIs*
	Male per 2000kcal	Female per 2000kcal	
**Qualifying nutrient**			
**Macronutrient**			
**Protein (g)**	57.8	48.9	RNI
**Dietary fiber (g)**	22.2	22.2	SPL
**Vitamin**			
**A (μg RAE)**	711.1	622.2	RNI
**B_1 (mg)**	1.24	1.07	RNI
**B_2 (mg)**	1.24	1.07	RNI
**B_{12} (mg NE)**	13.3	10.7	RNI
**C (mg)**	88.9	88.9	RNI
**D (μg)**	8.9	8.9	RNI
**E (mg α-TE)**	12.8	12.8	AI
**Mineral**			
**Ca (mg)**	711.1	711.1	RNI
**Fe (mg)**	10.7	17.8	RNI
**K (mg)**	1777.8	1777.8	AI
**I (μg)**	106.7	106.7	RNI
**Zn (mg)**	11.1	6.7	RNI
**Se (μg)**	53.3	53.3	RNI
**Disqualifying nutrient**			
**SFA** (g)**	22	22	< 10% E
**Added sugar (g)**	50	50	< 10% E
**Na (mg)**	1777.8	1777.8	UI

*NRD: Nutrient-Rich Diet Score; RDA: reference daily values; MRV: maximum reference values; DRIs: dietary reference intakes; RNI: Recommended Nutrient Intake; SPL: Specified Proposed Levels; AI: Adequate Intake; UI: Tolerable Upper Intake Level; E: energy intake.

**SFA: Saturated Fatty Acid.

Table S3. The percentage of ultra-processed foods and drinks consumption from 1997 to 2011

Survey Round	Male participants			Female participants		
	UPFDs (% per 2,000 kcal)	UPFs (% per 2,000 kcal)	UPDs (% per 2,000 kcal)	UPFDs (% per 2,000 kcal)	UPFs (% per 2,000 kcal)	UPDs (% per 2,000 kcal)
1997	4.10	2.61	1.48	3.05	2.93	0.12
2000	4.81	3.16	1.65	3.74	3.56	0.17
2004	5.39	3.12	2.27	2.95	2.73	0.22
2006	8.03	4.56	3.47	4.44	4.10	0.34
2009	8.21	5.15	3.05	4.92	4.53	0.38
2011	10.13	7.40	2.74	7.19	6.64	0.56

UPFDs: ultra-processed foods and drinks; UPFs: ultra-processed foods; UPDs: ultra-processed drinks.

Table S4. The average nutrient quality per 1,000 kcal of foods and drinks by degree of processing in the CHNS[1].

	n	Energy	Protein	Dietary fiber	SFA [2]	Added sugar	Sodium
		kcal/100g	g/1,000 kcal				mg/1,000 kcal
**All**	2,435	167.9±0.5	34.01±0.12	6.85±0.06	36.21±3.31	7.33±0.12	2,016.02±23.22
**Foods**	2,224						
Unprocessed and minimally processed (MPFs)	1,263	130.0±0.6	41.00±0.16	8.69±0.08	2.15±0.08	4.38±0.08	456.08±9.15
Processed culinary ingredients	158	368.0±2.3	16.85±0.19	3.45±0.05	5.57±0.05	3.56±0.14	4,027.82±66.10
Processed	193	164.0±2.4	70.91±0.92	7.20±0.24	9.45±0.18	1.89±0.24	1,155.61±24.69
Ultra-processed (UPFs)	610	210.6±2.4	33.43±0.38	7.03±0.10	1.33±0.05	34.30±0.52	5,477.92±19.09
**Drinks**	211						
Unprocessed or minimally processed (MPDs)	35	10.8±1.5	36.11±0.6.48	17.59±10.19	0	21.30±3.70	547.27±79.64
Processed	14	71.6±7.6	17.74±2.23	0	0	174.64±28.76	44.83±6.42
Ultra-processed (UPDs)	162	93.9±3.8	4.26±0.21	0.11±0.01	0	37.38±2.13	135.79±7.77

[1]CHNS: China Health and Nutrition Survey; [2]SFA: saturated fatty acid.

Table S5. Mediating effect of UPFDs consumption on the association between NRD15.3, GHGE, TWU, LU, diet cost and survey round[1].

	NRD15.3 (based on nutrients /2,000 kcal)	GHGE (kg CO2-eq/2,000 kcal)	TWU (m3/2,000 kcal)	LU (m2/2,000 kcal)	Diet cost (CNY/d/2,000 kcal)
**Survey round[2]**	10.063***	0.040***	0.009***	0.269***	0.953***
**UPFDs (g/2,000 kcal)**	-86.933***	0.383***	1.124***	1.154***	8.831***
**Survey round[3]**	0.003***	0.003***	0.003***	0.030***	0.003***
**Total mediated effect**	-0.027	0.028	0.279	0.115	0.030

1 *** indicates p-value < 0.001; ** indicates p-value < 0.01; * indicates p-value < 0.05. Abbreviations: NRD15.3: Nutrient-rich diet index 15.3; GHGE: Greenhouse gas emission; TWU: Total water use; LU: Land use; CNY: Chinese Yuan; UPFDs: Ultra-processed foods and drinks.

2 Survey round was set as continuous variable in this model.

3 The coefficient was computed by UPFDs as dependent variable and survey round as independent variable.

Table S6. Coefficients of two-level mixed effect models for nutrient quality (NRD15.3), diet-related environmental impacts, and diet cost in sensitive analysis, CHNS 1997-2011[a].

	**NRD15.3**		**GHGE log$_e$ (kg CO_2-eq/2,000 kcal)**		**TWU log$_e$ (m^3/2,000 kcal)**		**LU log$_e$ (m^2/2,000 kcal)**		**Diet cost log$_e$ (CNY/d·2,000 kcal)**	
**Effects**	**Model 1**	**Model 2**	**Model 1**	**Model 2**	**Model 1**	**Model 2**	**Model 1**	**Model 2**	**Model 1**	**Model 2**
Fixed effects (level 1)										
**Intercept**	6.767***	6.876***	0.762***	1.029***	1.216***	1.349***	0.963***	1.239***	1.664***	1.136***
**Survey round** (ref. =1997)[b]										
2000	-0.014	-0.002	-0.009	0.002	-0.022	-0.015	-0.037*	-0.028	Not measured	
2004	0.051***	0.040***	0.134***	0.094***	-0.032	-0.074***	0.054**	0.017	As ref.	
2006	0.079***	0.064***	0.188***	0.125***	0.031	-0.033	0.093***	0.030	0.083***	0.063**
2009	0.099***	0.075***	0.231***	0.146***	0.074***	-0.019	0.130***	0.047*	0.484***	0.445***
2011	0.140***	0.095***	0.278***	0.147***	0.142***	0.006	0.198***	0.068***	0.967***	0.930***
**UPFs** (log$_e$ (g/2,000 kcal))		0.002		0.012**		0.021***		0.026***		0.027***
**UPDs** (log$_e$ (g/2,000 kcal))		-0.012***		0.055***		0.050***		0.035***		0.182***
Age (per 10 years)		0.003		-0.024***		-0.016***		-0.022***		-0.003
Sex (ref. = male)		0.045***		0.009		0.032*		-0.002		0.028
**Lifestyle factors**										
Energy intake (per 100 kcal)		-0.004***		-0.009***		-0.009***		-0.009***		-0.008***
BMI (kg/m^2)		0.002**		0.003*		0.003*		0.004**		0.001
Activity level (ref. = Light)										
Moderate		-0.005		-0.017		-0.002		-0.019		-0.034
Heavy		-0.024***		-0.126***		-0.087***		-0.111***		-0.095***
Ever smoked (ref. = never smoke)		-0.019**		0.007		0.018		0.019		0.013
Time spent on Internet (per 10 minutes)		0.001*		0.004***		0.004***		0.004***		0.003**
**Socio-economic factors**										
Education attainment (ref. = Low)										
Medium		0.029***		0.042**		0.053***		0.045**		0.056**

High		0.013		0.052**		0.061**		0.071***		0.047
Income (per 1,000 CNY/month, inflated to 2011)		0.005***		0.007***		0.008***		0.006**		0.006**
Urbanicity (ref. = urban)		-0.053***		-0.135***		-0.105***		-0.144***		-0.124***
Random effects (level 2)										
Variance of Slope[c]	0.001	0.001	0.004	0.001	0.006	0.001	0.007	0.003	0.044	0.016
Variance of Intercept	0.003	0.005	0.052	0.046	0.078	0.029	0.099	0.027	0.899	0.226
Variance of Interaction	-0.001	-0.001	-0.001	-0.001	0.001	-0.001	0.001	-0.001	0.003	-0.001
Variance of Residual	0.022	0.020	0.065	0.061	0.067	0.065	0.071	0.070	0.090	0.084
Random-effects correlation coefficient	-0.996	-0.989	-0.037	-0.152	-0.690	-0.593	-0.651	-0.176	-0.666	-0.038
ICC[d]	0.120	0.201	0.444	0.430	0.540	0.309	0.582	0.278	0.909	0.729
AIC	3673.7	3471.1	3357.4	1528.3	3105.2	1452.6	3585.3	1767.1	4031.9	1922.0

[a] Abbreviations: NRD15.3: Nutrient-rich diet index 15.3; GHGE: Greenhouse gas emission; TWU: Total water use; LU: Land use; CNY: Chinese Yuan; UPFs: Ultra-processed foods; UPDs: Ultra-processed drinks; ICC: Inter-class correlation coefficient; AIC: Akaike information criterion. Level 1 represents the within-individual variations, which was assessed via the measure occasion; Level 2 represents the between-individual variations; NRD15.3, GHGE, LU, TWU, diet cost, UPD, and UPF were all transformed in Napierian logarithm form, and the NRD15.3 was calculated based on energy-standardized nutrient intake (2,000 kcal per day). Model 1: included measurements and individual variables; Model 2: added the Percentage of ultra-processed foods and drinks consumption as mediating variable, and covariates to Model 1. *** indicates p-value < 0.001; ** indicates p-value < 0.01; * indicates p-value < 0.05.

[b] For the cost of diet, the survey year is referenced to 2004.

[c] The random slope is combined by rooting the interaction terms of ln (UPFs) and ln (UPDs).

[d] The ICC is the proportion of the total variance that can be attributed to different trends between individuals.

Table S7. Proportion of Food consumption (%) in China from National Bureau of Statistics, 2013-2021* and in CHNS 2011.

Food group	CHNS 2011	2013	2014	2015	2016	2017	2018	2019	2020	2021
Cereal	35.3	41.0	39.4	37.8	36.8	36.1	35.5	34.9	36.2	35.0
Edible oils	2.9	3.0	2.8	3.1	3.0	2.8	2.8	2.7	2.6	2.7
Vegetable and fungus	27.3	27.0	27.1	27.5	27.7	27.5	26.8	26.6	26.7	26.6
Red meat	6.8	7.2	7.3	7.3	7.2	7.5	8.4	7.3	6.4	8.0
Poultry	1.5	1.9	2.2	2.2	2.5	2.5	2.5	3.0	3.3	2.9
Aquatic products	2.4	2.8	3.1	3.1	3.0	3.3	3.1	3.8	3.6	3.4
Eggs	2.8	2.2	2.5	2.8	2.8	2.8	2.8	3.0	3.3	3.1
Milk	2.5	3.3	3.6	3.4	3.3	3.3	3.4	3.5	3.3	3.4
Fruit	5.9	11.3	11.7	12.6	13.3	13.9	14.5	15.1	14.4	14.7
Sugar	0.2	0.3	0.3	0.3	0.3	0.3	0.3	0.3	0.3	0.2

*National Bureau of Statistics of China. https://data.stats.gov.cn/easyquery.htm?cn=C01
